# Comparative proteomic analysis of high cell density cultivations with two recombinant *Bacillus megaterium *strains for the production of a heterologous dextransucrase

**DOI:** 10.1186/1477-5956-4-19

**Published:** 2006-10-05

**Authors:** Wei Wang, Rajan Hollmann, Wolf-Dieter Deckwer

**Affiliations:** 1 Biochemical Engineering, Technical University Braunschweig, GBF/TU-BCE, Mascheroder Weg 1, D-38124 Braunschweig, Germany

## Abstract

High cell density cultivations were performed under identical conditions for two *Bacillus megaterium *strains (MS941 and WH320), both carrying a heterologous dextransucrase (*dsrS*) gene under the control of the *xylA *promoter. At characteristic points of the cultivations (end of batch, initial feeding, before and after induction) the proteome was analyzed based on two dimensional gel electrophoresis and mass spectrometric protein identification using the protein database "bmegMEC.v2" recently made available.

High expression but no secretion of DsrS was found for the chemical mutant WH320 whereas for MS 941, a defined protease deficient mutant of the same parent strain (DSM319), not even expression of DsrS could be detected. The proteomic analysis resulted in the identification of proteins involved in different cellular pathways such as in central carbon and overflow metabolism, in protein synthesis, protein secretion and degradation, in cell wall metabolism, in cell division and sporulation, in membrane transport and in stress responses.

The two strains exhibited considerable variations in expression levels of specific proteins during the different phases of the cultivation process, whereas induction of DsrS production had, in general, little effect. The largely differing behaviour of the two strains with regard to DsrS expression can be attributed, at least in part, to changes observed in the proteome which predominantly concern biosynthetic enzymes and proteins belonging to the membrane translocation system, which were strongly down-regulated at high cell densities in MS941 compared with WH320. At the same time a cell envelope-associated quality control protease and two peptidoglycan-binding proteins related to cell wall turnover were strongly expressed in MS941 but not found in WH320. However, to further explain the very different physiological responses of the two strains to the same cultivation conditions, it is necessary to identify the mutated genes in WH320 in addition to the known *lacZ*.

In view of the results of this proteomic study it seems that at high cell density conditions and hence low growth rates MS941, in contrast to WH320, does not maintain a vegetative growth which is essential for the expression of the foreign *dsrS *gene by using the *xylA *promoter. It is conceivable that applications of a promoter which is highly active under nutrient-limited cultivation conditions is necessary, at least for MS941, for the overexpression of recombinant genes in such *B. megaterium *fed-batch cultivation process. However to obtain a heterologous protein in secreted and properly folded form stills remains a big challenge.

## Background

*Bacillus *species are frequently used as bacterial workhorses in industrial microbial cultivations for the production of a variety of enzymes as well as fine biochemicals and antibiotics. The capacity of selected *Bacillus *strains to produce and secrete large quantities (20–25 g/L) of extracellular enzymes has placed them among the most important industrial enzyme producers. Indeed, they produce about 60% of the commercially available enzymes [1-4]. Compared with *B. subtilis*, the most studied model gram-positive bacterium, *B. megaterium *appears to be an attractive alternative host for recombinant protein production due to its intrinsic lack of alkaline proteases as well as the high stabilities in replication and maintenance of recombinant plasmids it hosts [5]. Therefore, foreign proteins from different sources have been chosen to explore the capability of *B. megaterium *as host bacterium for heterologous protein production, such as a dextransucrase (DsrS) from *Leuconostoc mensenteroides *[6,7], a levansucrase from *Lactobacillus reuteri *[8], and a hydrolase from *Thermobifida fusca *[9].

However, the yield obtained for these foreign proteins in secreted forms are often not satisfying. Especially DsrS, a big enzyme with a calculated molecular weight of 170 kDa, was found to be hardly secreted either in batch or fed-batch cultivations [6,7]. In general, high production of heterologous proteins in *Bacillus *hosts in secreted forms remains a challenge. The yields are reported to be 2 to 3 orders of magnitude lower than for native proteins or homologous proteins of closely related *Bacillus *species [1,4]. As already evidenced by numerous recent research works it is conceivable that secretion of heterologous proteins can be hampered at different stages of the secretion process, such as poor targeting of heterologous protein precursors to the membrane translocase, resulting in accumulation in the cytoplasm, or limitations in folding and stability of heterologous proteins at the membrane-cell wall interface which render those proteins vulnerable to attacks by wall-associated proteases, or critical interaction of heterologous proteins with the cell wall matrix components, as well as degradation of secreted heterologous proteins by extracellular proteases in the medium.

Therefore, it is important to gain insight into the physiological responses of the host cells to the specific cultivation conditions as well as to the induced production of heterologous proteins. Proteomic analysis has been evolved as a useful method that provides valuable information about the physiological state of microbial hosts under bioprocess conditions. Comprehensive analysis of protein expressions of cells at different time points throughout a given cultivation process can help to understand the adaptation of cells to potential environmental stresses and to discover metabolic bottlenecks of a production strain which can in turn help to optimize industrial production strains and processes [10-12]. A proteome analytic method based on two-dimensional polyacrylamide gel electrophoresis in combination with mass spectrometric protein identification (2-DE/MS) has been established in our laboratory and successfully applied for the investigation of protein expressions during heterologous dextransucrase production in batch cultivations with the *B. megaterium *strain MS941 [7]. In addition, after the availability of the genome sequence of the wild-type *B. megaterium *strain, DSM319, a strain-specific protein database has been created [13], which facilitate further our 2-DE/MS based proteomic analysis.

In this study, the standard technique to achieve high productivity, i.e. high cell density fed-batch cultivation was applied to further investigate the expression and secretion of the heterologous DsrS with two recombinant *B. megaterium *strains, a defined mutant MS941 and a chemical mutant WH320. As the result, the two strains demonstrated similar growth behavior but surprising difference in DsrS production. While WH320 produced DsrS with a maximal volumetric activity of 71700 U/L but without any detectable secretion in the culture medium, MS941 failed to express DsrS at all. Through comparative proteomic analysis certain differences in protein expressions were revealed in various cellular pathways, especially those involved in protein synthesis and secretion processes, as well as those in cell wall metabolism.

## Results and discussion

### Characterization of fed-batch cultivations for heterologous dextransucrase production with two recombinant *B. megaterium *strains

The establishment of a high cell density cultivation for *B. megaterium *and its use for the production of a heterologous dextransucrase (DsrS) from *Leuconostoc mesenteroides *has been described previously [6]. In this study a further improvement of DsrS yield was achieved with the strain WH320, but significant differences in the production of DsrS were observed between strain WH320 and strain MS941.

**Figure 1:**
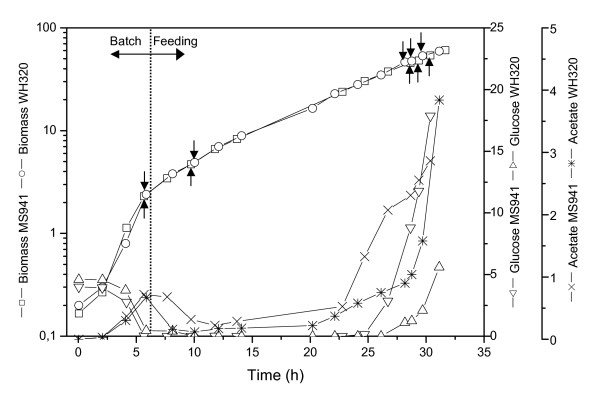
Profiles of biomass production, glucose consumption and acetate formation given in g/L during high cell density cultivations of *B. megaterium *strains MS941 and WH320. Arrows indicated the time points at which sample were taken for 2-DE analysis, namely at the end of batch phase (Batch), 4 h after the start of glucose-limited fed-batch phase (Feed), as well as at high cell densities shortly before induction (Ind0), 0.5 h after induction (Ind0.5) and 1.5 h after induction (Ind1.5).

The biomass, substrate and metabolite time courses of the high cell density cultivations with MS941 and WH320 are depicted in Figure 1. Both cultivations showed almost identical cell growth behavior, although acetate formation and glucose accumulation differed in the late feeding phase. The growth rate was approximately 0.12 h^-1 ^for both strains in the fed-batch phase, but decreased to 0.11 h^-1 ^for MS941 shortly before the induction of DsrS production with xylose. After induction, the growth rate reduced for both strains. WH320 showed a significant change to 0.09 h^-1^, while MS941 dropped further to 0.1 h^-1 ^during the induction period.

For MS941 glucose concentration in the medium decreased to 0.03 g/L at the end of the batch phase and maintained at zero for nearly 20 hours during feeding, but rose significantly in the late stage of the cultivation due to overfeeding under reduced growth rate, reaching 8.8 g/L shortly before induction and increasing further to 17.8 g/L in the following 1.5 h induction phase. For WH320 glucose concentration in the medium decreased to a barely detectable level at the end of the batch phase and maintained approximately at this level for nearly 20 hours during feeding before it increased to 1.1 g/L shortly before induction and increased further to 2.1 g/L in the following 1.5 h induction phase, clearly less than found with MS941. This difference may imply limitations of metabolism on different levels.

The main overflow metabolite detected by HPLC analysis was acetate. As shown in Figure 1 accumulation of acetate was observed for both strains during the batch phase, but merely at levels less than 1 g/L. Then acetate concentrations decreased after entering the fed-batch phase and glucose limitation. It increased again slightly for both cultivations afterwards, showing at first a similar trend for both strains. After approximately 20 hours cultivation time, acetate concentration started to rise for both strains, with a significantly higher rate for MS941. This was probably due to lack of trace elements in the culture with MS941, since adding trace elements shortly before the induction retarded the acetate production rate for MS941 during the induction phase, whereas an accelerated formation of acetate was detected for WH320 without the additional supplement of trace elements. While the increase in acetate concentration for MS941 prior to induction is substantial, the growth rate is not retarded significantly compared to WH320, thus there is at most only a slight inhibitory effect of acetate at these concentrations on MS941 growth. The general increase in acetate formation indicated an inability to fully oxidize the substrate in the latter part of the high cell density cultivation for both strains. Whether this is due to some sort of nutrient limitation, like the trace element shortage partially responsible for MS941 acetate formation, or an effect of quorum sensing or increasing heterogeneities within the environome of the bioreactor such as areas of low oxygen concentration, is unclear at present.

Table 1 shows the time course and volumetric activities of recombinant DsrS production in high cell density cultivation with WH320. The volumetric activity reaches a transient maximum of 71700 U/L 1.5 h after induction start. This corresponds to a specific activity of 1340 U/g_biomass_, a 3.7 fold increase over our previously published results [6]. Since most cultivation conditions applied are similar in these two HCDC cultivations, the overall increase in activity is most likely due to the addition of extra trace elements and MgSO_4 _at regular intervals of 7 hours in the new cultivation, while trace elements were added only at the beginning of the fed-batch and shortly before induction in the previous study [6]. The volumetric activity reduced to 47422 U L^-1 ^3 h after induction start. This is likely due to cellular responses such as increased proteolytic activity to the accumulation of misfolded and aggregated DsrS. Similar to the previous study, all activities detected are cell-associated. No secretion of DsrS into the culture medium was found. In addition, only a very low amount of 400 U L^-1 ^was soluble protein, the vast majority of activity was located in the insoluble cell fraction.

**Table 1:** Dextransucrase (DsrS) production during a high cell density fed-batch cultivation.

Time after induction (h)	Activity of DsrS (U/L)		
	
	cell-associated		Secreted
	
	soluble fraction	insoluble fraction	
0.5	157	30690	n.d.
1.5	415	71700	n.d.
3.0	365	47422	n.d.

Dextransucrase (DsrS) production during a high cell density fed-batch cultivation with *B. megaterium *strain WH320 carrying the plasmid pMM1520dsrS after induction with xylose, determined as volumetric activities of DsrS according to an activity staining method previously described [6-7].

n.d.: not detectable

In remarkable contrast to WH320, the induction of high cell density cultivation of MS941 resulted in no detectable activity of DsrS in any form. This lack of activity has been corroborated by a number of MS941 high cell density cultivations with induction of DsrS production (unpublished result). The lack of detectable DsrS activity in MS941 is puzzling. An inhibition by relatively high acetate concentrations is unlikely, as WH320 is well able to produce high amount of DsrS at similar acetate levels.

### Comparative proteomic analysis of cell physiological response to the fed-batch cultivation conditions and to recombinant dextransucrase production

As described above, if only the biomass production was compared, both *B. megaterium *strains demonstrated very similar cell growth behavior. But differences in glucose catabolism were evolved in the late feeding phase, accompanied by an unexpected difference in the expression of the heterologous DsrS. In order to gain a more comprehensive view on cell responses of the two strains to the given cultivation conditions as well as to find possible reason(s) for the differences in DsrS expression between the two strains, quantitative and comparative proteomic analysis was carried out by 2-DE analysis. Protein spots were identified using mass spectrometric methods such as MALDI-TOF MS and ESI-QqTOF MSMS and the specific *B. megaterium *protein database "bmegMEC.v2" [13]. As indicated in Figure 1, five samples were taken at different time points during the cultivation for each strain, namely at the end of the batch phase when glucose was consumed (Batch), in the early fed-batch phase under strict glucose limitation (Feed), in the late fed-batch phase at high cell densities shortly before induction (Ind0), 0.5 h after induction (Ind0.5) and 1.5 h after induction (Ind1.5).

**Figure 2:**
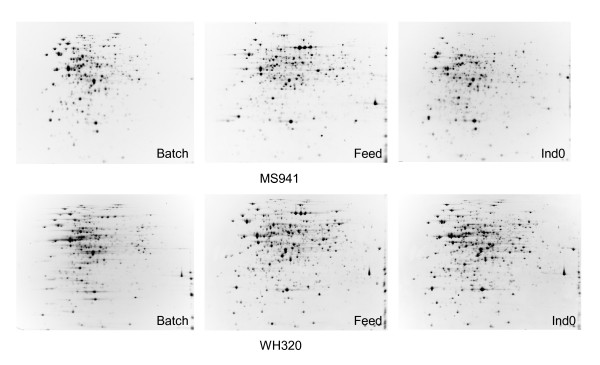
Two dimensional polyacrylamide gel electrophoretic analysis of intracellular (cell-associated) protein samples taken at different time points during high cell density fed-batch cultivations with *B. megaterium *strains MS941 and WH320. Description for samples is given in Figure 1 as well as in the text.

Shown in Figure 2 are images of the 2D PAGE gels obtained from intracellular (cell-associated) protein samples taken at the first three sampling time points (Batch, Feed and Ind0) both for MS941 and WH320. While the protein expression patterns are clearly different between these first three samples, they demonstrate less variation in the samples taken in the late fed-batch phase at high cell densities (Ind0, Ind0.5 and Ind1.5) within a strain, indicating that induction of DsrS production did not have noticeable impact on the metabolism and physiological states of the cells. One of the most extraordinary differences was the transient high-level expression of a homologous protein of the zinc-binding metalloprotease of the type immune inhibitor A (InhA) in the early fed-batch phase under strictly glucose limited conditions. InhA is normally associated with pathogenic *Bacillus *species as a virulence factor. This observation is published separately to this work [14]. Differences in protein expression pattern can also be visualized between the two strains, which is highly interesting in as far as it may permit an exploration of the different responses of the two strains to the high cell density cultivation conditions and to DsrS production at the proteome level. Protein spots whose expression levels revealed more than 2-fold variations between different samples within a strain as well as between the two strains were subjected to mass spectrometric (MS) analysis and resulted in the identification of proteins that are involved in different cellular pathways such as central carbon metabolism, overflow metabolism, amino acid synthesis and metabolism, protein synthesis, protein secretion and degradation, nucleotide metabolism, DNA/RNA processing, cell division and sporulation, cell wall metabolism, membrane transport, in general or specific stress responses. Here we will just present and discuss some of the results, especially those that will provide an overview in the different metabolic and physiological responses of the two *B. megaterium *strains to the cultivation and induction conditions applied. Such differences could possibly be related to the drastic difference in the expression of the heterologous DsrS.

### Central carbon metabolism

**Figure 3:**
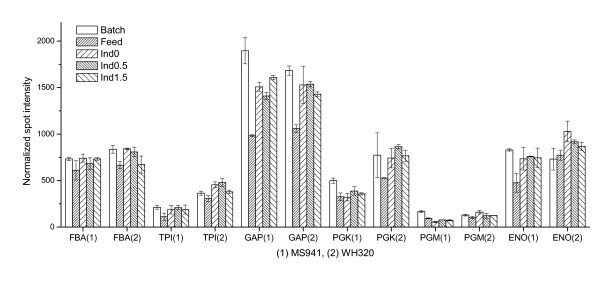
Expression profiles of some glycolytic enzymes during high cell density cultivations with strain MS941 (1) and strain WH320 (2). FBA: fructose 1,6-bisphosphate aldolase; TPI: triose phosphate isomerase; GAP: glyceraldehyde 3-phosphate dehydrogenase; PGK: phosphoglycerate kinase; PGM: phosphoglycerate mutase; and ENO: enolase.

As depicted in Figure 3, during the whole sampling period and for both *B. megaterium *strains, many glycolytic enzymes (FBA, TPI, GAP, PGK, PGM, ENO) showed similar expression profiles. This is conceivable since it is well known that some glycolytic enzymes form noncovalent multienzyme complexes within the cell and intermediates are channeled between them without being released into the surrounding medium. Therefore, expressions of these enzymes should be under tight and coordinated regulation. It has been reported that cultures deprived of glucose in comparison with cultures supplemented with glucose demonstrated reduced expression levels of glycolytic enzymes in *B. subtilis *[15] and *B. licheniformis *[16]. Comparing the levels of these glycolytic enzymes in the first two samples (Batch and Feed), both were under glucose-limited conditions, a slight decrease could be observed for most of these enzymes, indicating repressed glycolytic activities accompanied by successive degradations of these enzyme due to protein turnover during the early fed-batch process. In the later fed-batch process as glucose limitation was liberated and cells encountered an oversupply of glucose due to reduced growth rate in the last three sampling time points, up-regulation of the expression level could be observed to some extent for most of these glycolytic enzymes. For most glycolytic enzymes no obvious expression differences could be determined between the two *B. megaterium *strains, although accumulation of glucose was more pronounced in the cultivation with MS941 than that with WH320 in the later fed-batch phase (Figure 1). This suggests that a minor increase of glucose concentration in the culture medium can already trigger a reactivation of the glycolytic enzymes. However, it seems that expression levels of these glycolytic enzymes were, in general, not significantly varied but were maintained at relatively high levels also under glucose-limited cultivation conditions.

**Figure 4:**
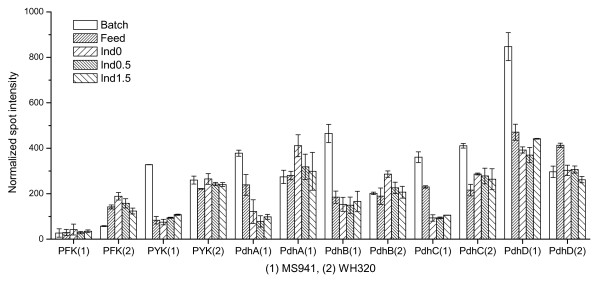
Expression profiles of phosphofruktokinase (PFK), pyruvate kinase (PYK) and pyruvate dehydrogenase complex (PdhA, PdhB, PdhC and PdhD) during high cell density cultivations with strain MS941 (1) and strain WH320 (2).

In contrast, two glycolytic enzymes, phosphofruktokinase (PFK) and pyruvate kinase (PYK), revealed certain differences in their expressions between MS941 and WH320 (Figure 4). Expression level of PFK was very low for MS941 in the whole sampling period, whereas it increased for WH320 during the fed-batch cultivation and was about 3 to 4-fold higher than for MS941. PFK is the major regulatory enzyme of the glycolytic pathway. The essentially irreversible reaction catalyzed by PFK is the step that commits a cell to channeling glucose into glycolysis. Up-regulation of PFK in fed-batch phase for WH320 probably signaled reduced ATP levels in the cell due to either a higher consumption of ATP or a lower generation rate of ATP. The reason for the generally lower PFK level in the culture with MS941 is not clear.

**Figure 5:**
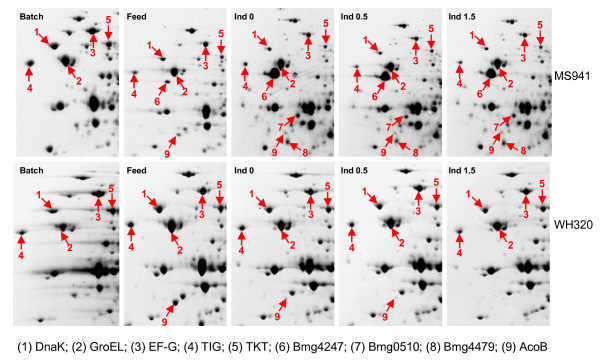
Sections of 2-DE PAGE gel images for the visualization of expression changes of some proteins. DnaK: heat shock 70 kDa chaperone DnaK; GroEL: 60 kDa chaperonin GroEL; EF-G: elongation factor G; TIG: trigger factor; TKT: transketolase; Bmg4247 and Bmg0510: peptidoglycan-binding proteins; Bmg4479: HtrA-type membrane-bound quality control protease; AcoB: acetoin dehydrogenase E1 component.

Pyruvate kinase (PYK) demonstrated a nearly constant expression level for WH320 during the whole sampling time period but a clearly reduced expression level in the fed-batch phase for MS941 (Figure 4). Similar to PYK expression profiles all the subunits of the downstream pyruvate dehydrogenase complex (PdhA, PdhB, PdhC and PdhD), were clearly down-regulated in the fed-batch phase for MS941, whereas most of them remained relatively constant for WH320 (Figure 4). Furthermore, differences were also observed between the two strains in the expression patterns of some enzymes involved in the pentose phosphate pathway. For instance, transketolase (TKT), a key enzyme involved in the non-oxidative PP pathway to create a bridge between glycolysis and the PP pathway, showed expression patterns similar to PYK and PDH complex, namely relatively constant expression in WH320 but reduced expression in MS941, as can be visualized on the sections of the 2-D PAGE gel images (Figure 5).

**Figure 6:**
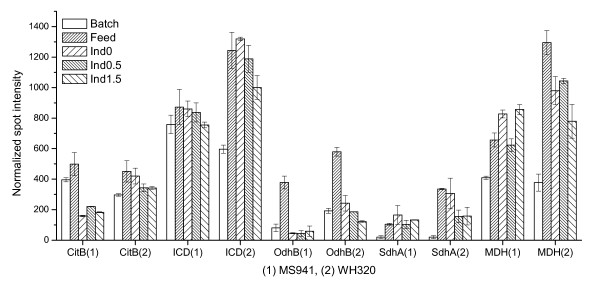
Expression profiles of some enzymes of the tricarboxylic acid cycle during high cell density cultivations with strain MS941 (1) and strain WH320 (2). CitB: aconitase; ICD: isocitrate dehydrogenase; OdhB: dihydrolipoamide succinyltransferase component of 2-oxoglutarate dehydrogenase complex; SdhA: succinate dehydrogenase flavoprotein subunit; MDH: malate dehydrogenase.

Comparing expression levels of the first two samples (Batch and Feed), enzymes involved in the tricarboxylic acid cycle (TCA cycle), such as CitB, ICD, OdhB, SdhA and MDH showed more or less up-regulated expressions (Figure 6). They should be mainly resulted from a derepression effect triggered by glucose limitation [15]. Thus, the down-regulation of glycolytic enzymes (Figure 3) and up-regulation of enzymes from TCA cycle during the early fed-batch phase – and probably also in the following fed-batch phase under glucose limitation – indicated that glucose supply was not sufficient to sustain the cell growth. Consequently, cells attempted to utilize alternative carbon and energy sources such as acetate, as evidenced by the reduced acetate concentration within this time period (Figure 1). In the later fed-batch phase expression changes showed no clear trends. However, higher expression levels were, in general, observed for WH320.

In conclusion, the two *B. megaterium *strains, MS941 and WH320, though both are derived from the same wild-type strain DSM319, demonstrated certain differences in the central carbon metabolism under the specific fed-batch cultivation conditions. Compared with WH320 the generally lower levels of some enzymes such as PYK, PDH complex, TKT and enzymes of the TCA cycle in the fed-batch phase for MS941, especially at high cell densities, might be a reason for the early and pronounced appearance of overflow metabolism indicated by the early and strong increase in acetate concentration and by the reduced growth rate shortly before the induction of DsrS production with xylose. However, the reason for the reduced expression levels or probably enhanced turnover rates of these enzymes in the cells of MS941 remains elusive.

### Overflow metabolism

Both MS941 and WH320 utilized not only acetate but also poly-β-hydroxybutyrate (PHB) and acetoin as alternative carbon and energy sources to sustain cell growth in the early fed-batch phase. Alterations on the expression levels of proteins that participate in the overflow metabolism gave evidences of the metabolic switches.

**Figure 7:**
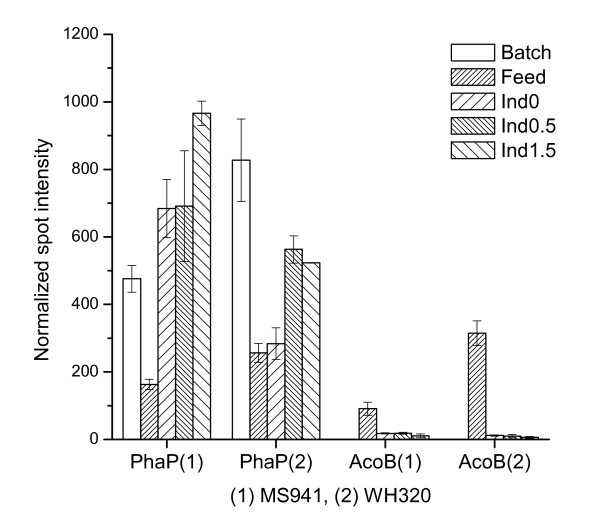
Expression profiles of enzymes involved in overflow metabolisms. PhaP is a PHB production-related protein; AcoB (acetoin dehydrogenase E1 component) is involved in acetoin metabolism.

It is well known that *B. megaterium *can produce PHB as an intracellular carbon and energy storage material. PhaP (Phasin) is a PHB production-related protein. It forms a boundary layer on the PHB surface to sequester hydrophobic PHB from the cytoplasm, thus inhibiting individual granules from coalescing and promoting PHB synthesis by the regulation of the ratio of surface area to volume of PHB granules [17,18]. PHB acts as an inducer for *phaP *expression, i.e. PhaP is only synthesized when the cells accumulate PHB [19,20]. In return, expression profile of PhaP can be an indication of PHB accumulation or dissipation in the cells. In our previous study accumulation of PhaP as one of the most abundant proteins of *B. megaterium *was observed in a batch cultivation with MS941 when glucose was not limited [7]. In this study during the early fed-batch process a strong reduction of PhaP levels were found both for MS941 and WH320 under glucose-limited conditions (Figure 7), implying that PhaP, and PHB as well, which first accumulated during the batch phase under unlimited growth condition was consumed by the cells as an alternative carbon and energy source to sustain cell growth. Afterwards, in the late fed-batch process glucose accumulated due to overfeeding, especially for MS941. As a result, re-accumulation of PHB and consequently, re-accumulation of PhaP took place for both strains, but was more pronounced for MS941.

A transient significant up-regulation of the acetoin dehydrogenase E1 component (AcoB) was detected for samples taken at the early stage of the fed-batch phase (Feed) for both strains under glucose limitation, especially for WH320. Then, expression levels were strongly down-regulated as glucose concentration increased in the culture medium due to overfeeding (Figure 5, Figure 7). Genes of the *acoABCL *operon are involved in the metabolism of acetoin, a prominent carbon overflow product in *B. subtilis*. They are recommended as marker genes for monitoring glucose availability in *B. subtilis *[11,15]. Interestingly, although the first samples taken at the end of the batch phase were also under prevailing glucose limitation, no AcoB was detected on the corresponding 2-D PAGE gels (Figure 5). It seems that at least in the *B. megaterium *strains used in this study the activation of the *acoB *gene in response to the exhaustion of glucose was delayed or there was still a barely detectable amount of glucose remained at this time point that was sensed by the cells and the *acoABCL *operon is activated only under strict glucose limitation.

### Protein synthesis and processing

**Figure 8:**
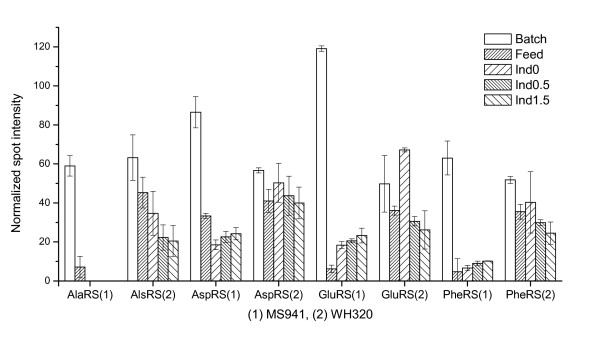
Expression profiles of some aminoacyl-tRNA synthetases for protein synthesis. AlaRS: alanyl-tRNA synthetase; AspRS: aspartyl-tRNA synthetase; GluRS: glutamyl-tRNA synthetase; PheRS: phenylalanyl-tRNA synthetase.

Protein synthesis takes place in several steps. In the first step each amino acid is activated and covalently attached to the corresponding tRNA by its specific aminoacyl-tRNA synthetase at the expense of ATP. Expression profiles of several such specific aminoacyl-tRNA synthetases (AlaRS, AspRS, GluRS, PheRS) are depicted in Figure 8. In general, they demonstrated both for MS941 and WH320 the highest levels at the end of the batch phase, which should reflect the high expressions of these aminoacyl-tRNA synthetase during the previous exponential cell growth in the batch phase. Afterwards, the aminoacyl-tRNA synthetase levels were sharply down-regulated for MS941 but remained remarkably higher for WH320 during the whole fed-batch process. This might indicate a clearly lower ATP level in the cells of MS941 than of WH320. Similar differences between MS941 and WH320 were also observed for the expression of enzymes related to many amino acid synthesis and metabolism, such as AroA, IlvB, IlvC and IlvD, as well as enzymes involved in nucleotide metabolism such as PurA, PurB, PurC and GuaA.

**Figure 9:**
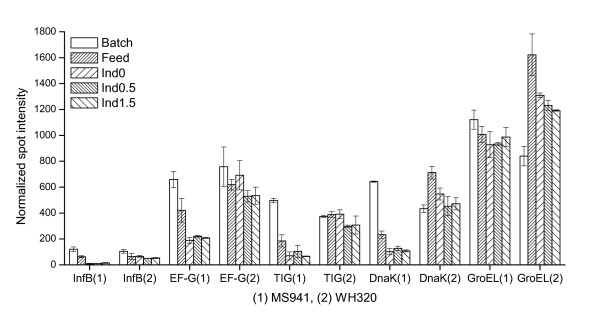
Expression profiles of some proteins implicated in protein synthesis. InfB: initiation factor IF-2; EF-G: elongation factor G; TIG: trigger factor. DnaK: heat shock 70 kDa chaperone DnaK; GroEL: 60 kDa chaperonin GroEL.

In the second step of protein synthesis several essential initiation factors are required for the initiation of translation. One such initiation factor, initiation factor IF-2 (InfB), which facilitates the binding of formylmethionyl-tRNA to the 30S ribosomal subunit during the translation initiation process, was identified on the 2-D PAGE gels. While InfB expression retained at a quite constant level in the whole fed-batch process for WH320, a significant down-regulation of InfB was detected for MS941 in the late fed-batch process (Figure 9). This strong reduction already happened before the induction of DsrS production (Ind0) and was, therefore, not resulted from the induction. Furthermore, in the cultivation with WH320 in which DsrS expression was detected, no noticeable drop of InfB level was found after the induction. Similarly, proteins involved in the following elongation step, such as elongation factor Tu (EF-Tu) and elongation factor G (EF-G) revealed distinctively lower expression levels within the same cultivation period for MS941 compared with WH320, as exemplified in Figure 9 for the expression profile and in Figure 5 for the change of the corresponding spot on the 2-D PAGE gels. Thus, expressions of proteins involved in the different steps of protein synthesis were all remarkably down-regulated for MS941 in the fed-batch phase, especially shortly before the induction as well as during the induction phase. The results suggest that compared with WH320 functions of many components of the cell's machinery for protein synthesis were diminished even before the induction of DsrS production. This could be an important reason that no DsrS was detected to be produced by MS941, whereas as many as 1340 U/g_biomass _were produced by WH320.

It has been reported that microbial hosts such as *E. coli *and *B. subtilis *respond to strong overproduction of heterologous proteins by elevated expression levels of some chaperone proteins like the heat shock 70 kDa protein DnaK, the 60 kDa chaperonin GroEL and the trigger factor TIG [21,10,11]. In the cytoplasm these chaperones have functions of preventing inappropriate aggregation or misfolding of newly synthesized proteins and promoting the proper assembly of unfolded proteins, or maintaining proteins destined for export in an open conformation during their translocation through the cell membrane. As shown in Figure 9 and Figure 5, expression levels of DnaK and TIG were obviously reduced in the fed-batch phase for MS 941 but remained at relatively constant levels for WH320. The reduced DnaK and TIG levels for MS941 may support the view of an essentially impaired function of protein synthesis and processing in MS941. For WH320 except a transient increase of the GroEL level in the early fed-batch phase, expression levels of DnaK, TIG and GroEL were not elevated during the time when a short-term high expression of DsrS took place in WH320. This might indicate that although the majority of DsrS produced was found to be accumulated in the cytoplasm, its amount was probably still not high enough to induce heat-shock like responses of these general chaperones in the cytoplasm.

### Protein secretion via the SecA-dependent pathway and secretion stress responses

Most proteins destined for export are translocated across the membrane in more or less unfolded conformations that allow them to pass through the translocation channel of the Sec pathway. For instance, in *B. subtilis *the majority of secretory proteins (~160) have been predicted to carry the typical Sec-type amino-terminal signal peptides in their precursor protein and consequently they are exported from the cytoplasm via the Sec pathway [22-24]. SecA, the ATPase subunit of the preprotein translocase complex is the translocation motor of the SecA-dependent translocation apparatus and plays a central role in coupling the hydrolysis of ATP to the transfer of secretory proteins across the membrane.

**Figure 10:**
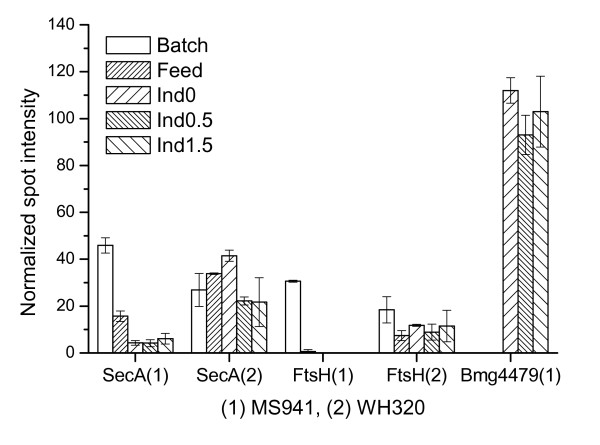
Expression profiles of the preprotein translocae SecA subunit, the membrane-anchored ATP-dependent protease FtsH and Bmg4479, a HtrA-type membrane-bound quality control protease.

As depicted in Figure 10, for WH320 SecA level was relatively constant within the experimental error range for the first three samples. It reduced then to a certain extent during the time of high-level DsrS expression (see Table 1). According to its signal peptide sequence DsrS is destined to be translocated via the SecA-dependent pathway. The result indicated that DsrS overexpression did not bring about a concomitant increase but rather, if any, a decrease in SecA expression. Similar to the general heterologous protein secretion problems associated with *Bacillus *species, significant accumulation of DsrS in the cytoplasm was identified for WH320 in high cell density cultivation [[6], and this work]. This could lead to secretion stresses at the cell membrane and might, in return, pose an obstacle to SecA expression. It has been reported that high-level expression of a heterologous α-amylase in *B. subtilis *resulted in a massive accumulation of the α-amylase precursor in the cytoplasm and did not lead to an increase in the amount of SecA [25].

A very different expression profile of SecA was observed for MS941. A clear decrease of SecA level was observed upon entering the fed-batch phase and this dropped continuously to a level about 10-fold lower in the late fed-batch phase at high cell densities. A comparable result has been reported for SecA expression in *B. subtilis*. Transcription of the *B. subtilis secA *gene was observed to occur mainly in exponential growth phase and peaked almost precisely at the transition to stationary phase, afterwards *de novo *transcription decreased sharply to a low basal level [25]. Consistent with the transcription results, intracellular level of SecA protein in *B. subtilis *was found to remain unchanged in exponential growth phase, irrespective of the overexpression of two native secretory proteins that was expected to cause potentially secretion stresses. SecA level then dropped after entering stationary phase and reached a basal level about 12-fold lower in the late stationary phase [26]. In our study if presumed that SecA level at the end of the batch phase (Batch) still reflected to some extent the expression of SecA during exponential growth phase, its sharp fall at high cell densities for MS941 should reflect a cellular physiological condition resembling the late stationary phase of a batch cultivation. Since the *xylA *promoter system employed for the heterologous *dsrS *gene expression is more suitable for gene expression during exponential-growth phase [6], it is conceivable that the cellular state of MS941 might not allow the initiation of *dsrS *gene expression. Consequently no DsrS could be detected in the fed-batch cultivation with MS941.

It is well known that one of the various possible post-exponential growth means by which *B. subtilis *and related Bacillus species adapt to unfavorable growth conditions is the increased protein secretion activity [2,26]. This is also true for *B. megaterium*. Generally fewer proteins could be found in the culture supernatants in exponential growth phase than those from stationary phase. The elevated secretion activity seems contradictory to the reduced SecA level in stationary phase. However, even translocation of native secretory proteins has been reported to be not always straightforwardly correlated with SecA level. For example, SecA level effected quite differently on the secretion of the native levansucrase and α-amylase in *B. subtilis*. Increase in SecA level resulted in an elevated production of extracellular levansucrease and decrease in SecA level led to reduced yield of secreted levansucrase with concomitant accumulation of unprocessed precursor in the cells, whereas α-amylase secretion was almost unaffected even at very low SecA levels. Therefore, it has been suggested that a basal level of SecA is perhaps sufficient to facilitate the translocation of most native secretory proteins, including those that are mainly produced and secreted as degrading enzymes when the cells encounter nutrient limitations [26]. Differences in protein secretion are explained as a likely consequence of different affinities of their precursor proteins for SecA. The overall hydrophobicity of α-amylase is much greater than that of levansucrase [26]. Later it has been suggested that in *B. subtilis *signal peptide hydrophobicity is critical for the early stage protein export and the *B. subtilis *protein export apparatus seems to be poorly adapted to handle alanine-rich signal peptides with low hydrophobicity [27]. In order to further employ *B. megaterium *for heterologous protein production, it would be very helpful to find out in the further study whether the *B. megaterium *protein export apparatus functions similarly to that of *B. subtilis*.

It is worth to mention that similar to SecA expression in *B. subtilis*, we have also observed an increase of the intracellular SecA level in the late exponential growth phase of batch cultivations with MS941, as well as a primary correlation between the increase in the SecA level and the increase of DsrS yield in secreted form (unpublished result). However, most DsrS produced was still either accumulated in the cytoplasm or retained in the membrane/cell wall interphase. Less than 2% DsrS was secreted into the surrounding medium in batch cultivations with MS941 [7]. No DsrS in secreted form was detected in high cell density fed-batch cultivation with WH320, as evidenced both in this and in a previous study [6]. Taking together the results of batch cultivation and high cell density cultivation, it is likely that regulation of SecA expression in *B. megaterium *is not directly influenced by potential secretion stresses resulting from DsrS overexpression. Rather, existence of a certain amount of SecA is the prerequisite for an effective translocation of this heterologous protein.

Once secretory proteins pass through the cytoplasmic membrane, they need to fold into their native conformations on the *trans *side of the membrane prior to their translocation through the cell wall and release into the culture medium. The rate and efficiency of folding are critical at this step, since unfolded, partially folded or incorrectly folded proteins are susceptible to proteolytic degradation. Normally native secretory proteins have evolved along with the secretion apparatus to have physico-chemical properties that facilitate their rapid and correct folding in the microenvironment of the cell envelope and enable a minimal retention by the cell wall matrix for proteins destined for truly extracellular locations [28,29]. In contrast, heterologous secretory proteins may not possess such physico-chemical properties and are much more susceptible to proteolytic activities of cell-associated proteases. Cell-associated proteolysis is a significant problem affecting the use of *Bacillus *species, such as *B. subtilis *as host bacterium for the production of heterologous proteins [30].

Accumulation of misfolded proteins at the membrane/cell wall interface induces the expression of genes encoding HtrA-type membrane-bound quality control proteases. These proteases degrade misfolded or unfolded proteins in the cell envelope under secretion stress conditions [31]. In *B. subtilis*, two members of this protease family, namely HtrA and HtrB, have been identified and studied extensively with respect to their roles to rescue cells from cell envelope-associated stresses in response to secretion or heat stresses caused by misfolded proteins [32-39]. Expression profiles of HtrA and HtrB has been reported to be induced by temperature upshift or by overproduction and secretion of heterologous proteins. Secretion stress is sensed, among others, by the autoregulated CssRS (control of secretion stress regulator and sensor) two-component system and transcription of *htrA *and *htrB *genes is strictly controlled by the CssRS system [34-36]. Two-component signal transduction system is an important mechanism by which bacteria sense the prevailing physical, chemical and nutritional conditions and transduce this information into the cells so that an appropriate response can be effected [40].

**Figure 11:**
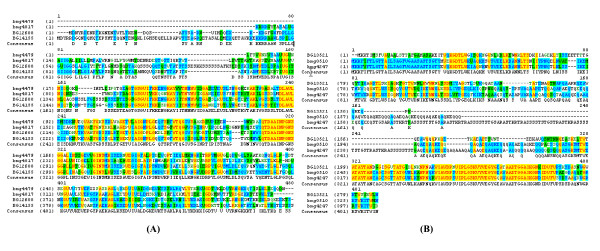
Multiple sequence alignments of (A) two HtrA-type membrane-bound quality control proteases Bmg4479 and Bmg4817 from *B. megaterium *and the HtrA (BSORF: BG12608) and HtrB (BSORF: BG14155) proteins from *B. subtilis*; (B) two peptidoglycan-binding proteins Bmg4247 and Bmg0510 from *B. megaterium *and the YocH (BSORF: BG13521) protein from *B. subtilis*.

As shown in Figure 5, a protein spot was identified to be a homologue of both HtrA and HtrB proteins from *B. subtilis*, showing 50% and 48% sequence identity, respectively. This protein is designated as Bmg4479 in the strain-specific protein database "bmegMEC.v2". We found another homologous protein presenting in "bmegMEC.v2" as Bmg4817 which has 43% and 42% sequence identity to the *B. subtilis *HtrA and HtrB proteins, respectively. The multiple sequence alignment shown in Figure 11a reveals sequence consensus in the middle region and in the C-termini. The sequence of Bmg4479 is apparently incomplete in its N-termini. Since the coding sequence is at the end of a contig, the missing part is obviously not yet sequenced. Thus, *B. megaterium *seems also to have two HtrA-type proteins. Bmg4817 was not among the protein spots identified in this study. Probably its expression was suppressed by Bmg4479, since it has been reported that transcriptions of the homologous genes *htrA *and *htrB *in *B. subtilis *are negatively cross-regulated [32,33].

Expression of Bmg4479 revealed a strong up-regulation in the late fed-batch phase at high cell densities in MS941 (Figure 10). Actually the corresponding spot was not found in the samples taken at the end of the batch phase or in the early fed-batch phase, as can be visualized on the 2-D PAGE gel images (Figure 5). Bmg4479 was already detected before the induction of DsrS production. This may reflect a cellular state of MS941 in which, due to still unknown reasons, even native protein secretion encountered secretion stresses under the high cell density conditions applied in this study. Consequently, the proteolytic activity was increased to deal with misfolding and/or aggregation of native secretory proteins in the cell envelope of MS941. Thus, under such circumstances if there was any DsrS expressed, it would have caused additional secretion stresses and therefore, damaged the viability of the cells of MS941. It is conceivable that cells might reduced the synthesis of proteins destined for secretion, including DsrS, as well as reduced the translocation of such proteins through the membrane, as indicated by the very low SecA level.

Interestingly, Bmg4479 was not identified in any samples of WH320. This result is quite surprising, since in contrast to MS941 a high-level expression but no secretion of DsrS was confirmed in the fed-batch cultivation with WH320, one would expect that such cell envelope-associated quality control proteases would be activated in the cells of WH320 to cope with the secretion stresses. Thus, despite the fact that DsrS was hardly released into the culture medium and cell-associated DsrS was confirmed to accumulate in the cell membrane/wall interphase, folding and stability of DsrS on the trans side of the membrane seems not to be the rate-limiting factors that would trigger a visible up-regulation of HtrA-type proteases. Another possible explanation can be that in the undefined chemical mutant WH320, except the known inactivation of the *lacZ *gene [41], other genes might be damaged as well. As the result, *bmg4479 *gene itself or genes that regulate the expression of *bmg4479*, such as – but not necessarily restricted to – the two-component CssRS system, are inactivated. Furthermore, it can not be excluded that in WH320 it was other yet unidentified cell envelope-associated proteases involved in degradation of the accumulated DsrS in the cell membrane/wall interphase.

FtsH is also a membrane-anchored ATP-dependent protease that has pleiotropic functions [42-44]. FtsH was shown to be involved in the degradation of uncomplexed SecY protein, an essential component of the preprotein translocase complex. Elimination of uncomplexed SecY is important for the integrity of the protein translocation machinery and an optimum protein translocation. In the absence of FtsH uncomplexed SecY accumulated to levels that are deleterious to protein export, cell growth and viability [42,43]. Because of its high *pI *value of 9.9, SecY was not detected on the 2-DE PAGE gels in the pH range of 4–7. However, the expression profile of FtsH demonstrated a drastic fall as the cultivation entered the fed-batch phase and became barely to detect at high cell densities for MS941, whereas for WH320 the drop of the FtsH level was not that significant and remained nearly constant during the whole fed-batch phase (Figure 10). It is therefore reasonable to suggest that the drastic decreased FtsH level might have certain negative effect on the functionality of the protein translocation channel. In *B. subtilis ftsH *gene expression was found to be maintained during vegetative growth but steadily declined as the cells entered the stationary phase of growth [44]. Likely, the drastic fall of FtsH level in MS941 at high cell densities again reflected a cellular state resembling late stationary phase of batch cultivation, which is inadequate for the aimed heterologous DsrS production.

In addition, *ftsH *mutants were found to be unable to enter the developmental life cycle under starvation conditions but underwent extensive cell lysis [44]. In our study the total protein concentrations in the culture supernatants shortly before the induction of DsrS production were determined to be more than 5-fold higher for MS941 than for WH320, namely 289 mg/L for the former and 46 mg/L for the latter, indicating a severe cell lysis of MS941 under high cell density conditions rather than an elevated secretion activity. The "deficiency" in FtsH of MS941 seems to reflect a similar cellular physiological state described for *B. subtilis ftsH *mutants.

### Cell wall metabolism

In their combined transcriptomic and proteomic analyses of *B. subtilis *fed-batch fermentation processes Jürgen et al. [12] observed in the late growth phase under high cell density and slow growth conditions a weak up-regulation of autolysis genes such as *lytC*, involved in cell separation and cell wall turnover; *cwlH*, related to mother cell lysis; and *blyA*, coding for a protein with N-acetylmuramoyl-L-alanine amidase activity. They further pointed out that the increased expression level of these genes could be a reason for the increased cell lysis under high cell density conditions, which is known to be a critical problem of industrial *Bacillus *fermentation processes.

**Figure 12:**
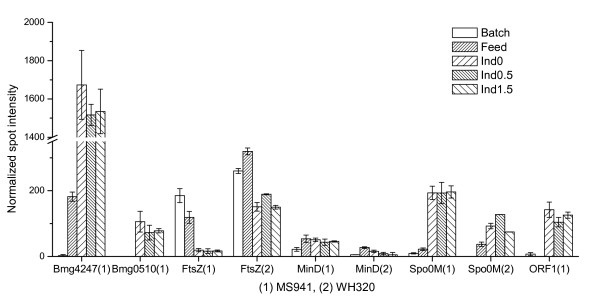
Expression profiles of enzymes involved in cell wall turnover, or cell division and sporulation. Bmg4247 and Bmg0510: peptidoglycan-binding proteins; FtsZ: cell division protein FtsZ; MinD: cell division inhibitor MinD; Spo0M: sporulation control protein; ORF1: sporulation related protein.

In the present study a remarkably up-regulated expression of a protein spot (Figure 5 spot 6) was found for MS941 on the 2-D PAGE gels from the sample taken shortly before the induction (Ind0). The expression level remained nearly constant during the following 1.5 h induction phase (Figure 12). Interestingly, appearance of this protein spot could not be detected on any of the 2-D PAGE gels for WH320. It was one of the proteins that revealed the highest expression levels under high cell density conditions of MS941 and demonstrated a most striking difference between the two *B. megaterium *strains. MALDI-TOF MS and ESI-QqTOF MS/MS analyses identified this protein, designated as Bmg4247 in the protein database "bmegMEC.v2", as a peptidoglycan-binding protein. In addition, another peptidoglycan-binding protein, Bmg0510, was also identified but again only for MS941 (Figure 5, spot 7). Bmg0510 demonstrated a similar expression profile, but its expression level was about 20-fold lower than Bmg4247 (Figure 12). Although Bmg4247 and Bmg0510 have only a sequence identity of 46%, the similarities of their N-terminal as well as C-terminal sequences are very high (Figure 11b). Analyzing protein domain structures of Bmg4247 and Bmg0510 by querying against the InterPro database, a database of protein families, domains and functional sites [45] revealed that both proteins possess a C-terminal domain typical of N-acetylmuramoyl-L-alanine amidase and a N-terminal peptidoglycan-binding LysM (lysin motif) domain. LysM domain is found in a variety of enzymes involved in bacterial cell wall degradation. A most likely cleavage site between pos. 24 and 25: ASA-ST is also predicted for both proteins by the signal peptide prediction program SignalP 3.0 [46].

BLAST searching for orthologous proteins of Bmg4247 and Bmg0510 against the "13 *Bacilli*" protein database, a database that contains protein sequences of the 13 *Bacillus *species whose complete genome sequences are currently available [13], leads to the identification of the most homologous protein being the *B. subtilis *YocH protein (Figure 11b). YocH is a autolysin-like cell wall-binding protein with a putative peptidoglycan hydrolase activity. Expression of the *yocH *gene in *B. subtilis *has been confirmed to be specifically activated by YycF of the YycG (histidine sensor kinase)-YycF (response regulator) two-component signal transduction system [47]. YycFG system has been proven to be essential for cell viability. It was first identified in *B. subtilis *and is well conserved in other low G+C Gram positive bacteria [48,49]. YycFG is suggested to play a role in cell division and in cell membrane/cell wall homeostasis. Genes such as *ftsAZ *for cell division, *yocH *and *ykvT *as autolysin/autolysin-like proteins for cell wall turnover, *and tagAB, tagDEF *for teichoic acid biosynthesis have been identified as prominent members of the YycFG regulon in *B. subtilis *[47,49-51]. YycFG system is also shown to play a role in controlling virulence and cell wall metabolism in *Staphylococcus aureus *and *Streptococcus pneunomiae *[52,53].

Searching for homologous proteins of the *B. subtilis *YycF and YycG proteins in our *B. megaterium *database "bmgMEC.v2" confirmed the presence of their orthologues as Bmg4821 and Bmg4822, showing sequence similarity of 55% and 83%, respectively. In addition to their corresponding coding genes *bmg4821 *and *bmg4822*, four additional genes, namely *bmg4817, bmg4818, bmg4819 *and *bmg4820*, were found within the same contig in the *B. megaterium *genome sequence. Proteins encoded by *bmg4817, bmg4818, bmg4819 *and *bmg4820 *are orthologues of the *B. subtilis *YycK, YycJ, YycI and YycH proteins, respectively. YycH, has been reported to be the repressor protein that regulates the activity of the YycFG system to keep optimum cell growth and cell wall levels. All the six genes, *yycF, yycG, yycH*, *yycI, yycJ *and *yycK *in *B. subtilis *belong to the essential yycFG two-component system [54]. The corresponding six genes in *B. megaterium *are also adjacent genes and are arranged in the same succession as their orthologues in *B. subtilis*. It is, therefore, evident that a similar YycFG two-component system exists also in *B. megaterium*.

Expression of *yocH *has been confirmed to be positively regulated by the phosphorylated form of YycF [47]. Consequently, YocH has been used as the prominent indicator whose expression level reflects the YycF activation level [51,54]. Therefore, the strong up-regulations of Bmg4247 and Bmg0510 found in this study might indicate a significant activation of YycF under the given high cell density cultivation conditions. Expressions of Bmg4247 and Bmg0510 were not visualized for samples taken at the end of the batch phase or at the early stage of the fed-batch phase (Figure 5). Checking our previous 2-DE experiment results of batch cultivations with MS941 during exponential growth, we could not find the corresponding protein spots on any of the 2D PAGE gel images. Expression of these two peptidoglycan-binding proteins in *B. megaterium *seems to be only associated with the high cell density conditions applied in this study. This result seems different to what have been reported for *B. subtilis*, namely in sporulation medium the YycG/YycF system is only present in the vegetative phase with levels decreasing rapidly at the onset of stationary phase. In accordance, expression of YycF-dependent genes is only activated during the exponential growth phase and stopped at the beginning of stationary phase [47,48]. However, in LB medium both YycG and YycF levels have been found to stay constant throughout the cell growth cycle [54]. Thus, it seems that YocH expression depends strongly on cultivation condition applied.

The fact that expressions of both Bmg4247 and Bmg0510 were strongly up-regulated as the cell density increased indicates probably that there was an elevated need on these cell wall-binding enzymes under high cell density conditions. Function of the orthologous *B. subtilis *YocH protein has not yet been characterized but is thought to be likely an autolysin related to the amidase domain of the major bifunctional *Staphylococcus aureus *autolysin ATL [55]. The *atl *gene products are suggested to be involved in the hydrolysis of septal peptidoglycan to facilitate the separation of dividing cells [56,57]. They are also implicated in cell wall turnover with *atl *mutant showing complete inhibition of metabolic turnover of cell wall peptidoglycan [58]. For cell wall turnover the inside-to-outside model of cell wall assembly in *B. subtilis *suggest that autolysins are necessary for the hydrolysis and remove of old stressed peptidoglycan to allow nascent peptidoglycan, which is laid down along the cytoplasmic membrane, to expand and become stress bearing as the cell elongates [59]. In addition, cell wall turnover is believed to be required for secretion of large proteins. The cross-linked covalent network of peptidoglycan in bacterial cell walls can present a physical barrier to protein secretion, which may be lessened by the action of autolysins [55]. Thus, under high cell density conditions the much higher extracellular protein concentration of MS 941 compared with that of WH320 might be correlated with an elevated cell wall permeability resulted from an unusual high cell wall turnover rate as indicated by the high expression levels of Bmg4247 and Bmg0510.

It remains to be elucidated why MS941 needed simultaneously two autolysin-like enzymes which seems functionally redundant, why these enzymes were not found in the proteome of WH320 which was cultivated under the same conditions. Again, it seems that the undefined chemical mutant WH320 was deficient in response to environmental signals that should trigger the gene expressions controlled by the YycFG two-component system.

It is worth to note that the time course of Bmg4247 expression level coincided with the change of the biomass concentration. Bmg4247 was first detected in the early fed-batch phase (Feed) with a biomass concentration of 4.7 g/L (Figure 12). Its expression level increased nearly 9-fold as the biomass concentration increased to 46 g/L shortly before the induction (Ind0) and remained, like the biomass concentration, relatively constant within the experimental error range during the following 1.5 h cultivation time. Like YocH in *B. subtilis*, if Bmg4247 can be also an indicator of the YycF activation level, the correlation between the Bmg4247 level and biomass might further indicate that biomass concentration might be an environmental signal that can indirectly activate the YycFG two-component system as a kind of "quorum sensing" response.

### Cell division and sporulation related proteins

The YycFG two component system has also been suggested to be involved in modulating the expression of the essential cell division operon, *ftsAZ *in *B. subtilis *[47,50]. Overproduction of the YycF regulator induces excess cell division. Expression of an essential cell division gene, *ftsZ*, is suggested to be potentially under the direct control of the YycFG system [50]. In our present study expression level of the cell division protein FtsZ encoded by the *ftsZ *gene was reduced after entering the fed-batch phase. It demonstrated further no up-regulation but rather a clearly down-regulation at high cell densities for MS941 (Figure 12). This was contrary to what would be expected as a result of a strongly elevated YycF activity in this late fed-batch phase for MS941. Probably the YycF level in MS941 is not comparable with the overproduced YycF level in *B. subtilis *to bring about a similar effect on cell division. Under high cell density conditions FtsZ level dropped as well but to a less extent for WH320. The decreased FtsZ level might reflect reduced cell division at high cell densities due to lower growth rates, although it has been reported that intracellular concentration of FtsZ remains constant irrespective of growth rates [60]. Another cell division related protein, the cell division inhibitor MinD, is required for the correct placement of the division site in *B. subtilis*. It acts in concert with another cell division inhibitor MinC to block the localization of FtsZ ring to the cell pole and inhibit polar division in vegetative cells. Expression level of MinD was up-regulated in the fed-batch phase for both strains and was generally higher for MS941 than for WH320, indicating probably a higher demand on this protein for the control of correct cell division in MS941.

Also shown in Figure 12 is the expression profile of Spo0M, a sporulation related protein. Expression of Spo0M was clearly induced after entering fed-batch phase for both *B. megaterium *strains. In the late fed-batch at high cell densities Spo0M level increased further, especially for MS941. In *B. subtilis *expression of the *spo0M *gene is confirmed to be under the control of σ^H^, an alternative sigma factor that regulates, among others, the transcription of genes that are induced in late-growth cultures at high cell density, including genes that function in sporulation [61,62]. Disruption of *spo0M *results in considerable impairment in sporulation, and the morphological stage blocked in sporulation is stage 0. However, overproduction of Spo0M exerts also certain negative effects on sporulation [63]. Furthermore, transcription of a quorum sensing peptide, the competence and sporulation factor (CSF), has been identified to be also controlled by σ^H ^and increased as cells entered stationary phase, contributing to the increase in extracellular CSF at this time [64].

ORF1 (Figure 12) has been reported being a gene of an operon that is expressed at late sporulation phases [65]. Transcripts of the *orf1 *gene has been found to accumulate at sporulation stage IV/V [66]. ORF1 was only detected for MS941 with strongly up-regulated expression under high cell density conditions. This result might indicate that MS941 were quite inhomogeneous under the given cultivation conditions, consisting of cells of different cellular development stages.

### Extracellular proteome (the secretome)

**Figure 13:**
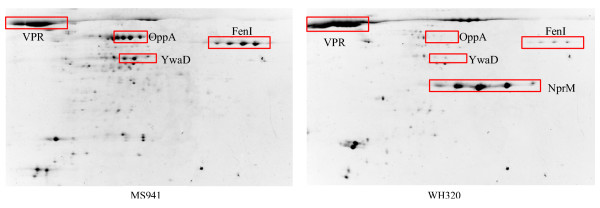
Two dimensional gel electrophoretic analysis of secreted proteins (secretome) in the culture supernatant taken in the early fed-batch phase (Feed) of high cell density fed-batch cultivations with *B. megaterium *strains MS941 and WH320. OppA: oligopeptide-binding protein; FenI: FenI protein (function unknown); YwaD: aminopeptidase; VPR: extracellular serine protease; NprM: neutral metalloprotease.

As expected, a high amount of the neutral metalloprotease NprM was detected for strain WH320 but not for MS941 (Figure 13), because the gene coding for NprM was disrupted in the strain MS941 [65].

OppA, the oligopeptide-binding protein of the Opp transport system, demonstrated a remarkable difference in the secretome between the two *B. megaterium *strains (Figure 13). OppA was found as an abundant protein in the secretome of MS941 both in the early fed-batch phase as well as in the late fed-batch phase at high cell densities (not shown). In contrast, it was barely detected in the secretome of WH320. The Opp system plays a very pleiotropic role since it is implicated in the uptake of various oligopeptides that act as, for example, cell density signals for the cells [67,68]. In *B. subtilis*, the Opp system imports extracellular peptides including CSF that influence cell competence development and sporulation, as well as antibiotic biosynthesis [69-71]. A major role of the Opp system in Gram-negative bacteria is suggested to be the recycling of cell-wall peptides as they are released from the growing peptidoglycan [72].

In addition, the Opp operons of *B. subtilis *and *S. aureus *have been identified as potential members of the YycF regulon. YycF could control the synthesis of the Opp system [47,52]. A DNA-motif analysis of the promoter region of OppA in the *B. megaterium *wild-type strain DSM319 using the program "Virtual Footprint" [73] reveals a DNA sequence that matches exactly the consensus recognition sequence for the YycF response regulator defined by Howell et al. [47]. Therefore, it is reasonable to suggest that at least in the *B. megaterium *strain MS941 under the given fed-batch cultivation conditions, the YycFG two-component system can also modulate the synthesis of the Opp system to satisfy the demand on the transport of signaling peptides probably released from the cell-wall by peptidoglycan hydrolases like Bmg4247 and Bmg0510. The lack of OppA in the secretome of WH320 was perhaps resulted from the lack of activity of the YycFG system, as has been already discussed above in relationship to the peptidoglycan-binding proteins Bmg4247 and Bmg0510.

Furthermore, Opp system is found to be required in the pathogenic *Bacillus *species *B. thuringiensis *and *B. cereus *for the expression of the plcR regulon at the onset of stationary phase by the uptake of a signaling peptide acting as a quorum-sensing effector. PlcR is a pleiotropic regulator of virulence factors. It activates the transcription of genes encoding extracellular proteins, including phospholipases C, proteases and enterotoxins [74]. Interestingly, homologous proteins of Bmg4247 in the *B. cereus *strains are assigned the function of enterotoxin, it is, therefore, tempting for us to speculate that a regulation circuit might exist as YycF controlling the synthesis of the Opp system and OppA in return playing a role in the induction of the expression of Bmg4247 and Bmg0510 through activating the YycFG two-component system.

Another obvious difference in the secretome between the two strains is the Bmg0280 protein. It presented a much higher secreted amount in MS941 than in WH320. Bmg0280 is homologous protein of the FenI proteins from *B. subtilis*, *B. cereus *and *B. thuringiensis *found in the UniProt database Swiss-Prot/TrEMBL, with 67%, 70% and 70% sequence identities, respectively. Consequently Bmg0280 is designated as the *B. megaterium *FenI protein. Function of FenI protein is still unknown. However, another *B. subtilis *YngK protein was found to be also a homologous protein showing 68% sequence similarity to Bmg0280. Since the FenI protein (Q9L7W7) and the YngK protein (O35015) of *B. subtilis *have a sequence identity of 82%, they are at least paralogous proteins with presumably similar functions. The *B. subtilis *YngK protein is suggested to be involved in the synthesis of an antifungal lipopeptide antibiotic, pliplastin. Mutation in YngK resulted in elevated activity of σ^X ^[75], an extracytoplasmic function (ECF) sigma factor that functions in regulating cell envelope modification as a defense against cationic antimicrobial peptides in *B. subtilis *[76]. Whether the *B. megaterium *FenI protein possesses a similar function needs to be elucidated.

The extracellular serine protease VPR was the most abundant secreted protein for both *B. megaterium *strains under nutrient starvations that was not necessarily restricted to glucose limitation (Figure 13). The extracellular serine protease (VPR) has been identified as a putative member of the PhoP regulon and is likely to be important for the recovery of inorganic phosphate from a variety of organic sources of phosphate in the environment. [77]. VPR has also been reported to be one of the extracellular serine proteases involved in the processing of the pentacyclic lantibiotic subtilin [78], which in turn has been proved to function as a pheromone for quorum sensing [79].

A homologue of the *B. subtilis *YwaD protein was also identified in the secretome of both *B. megaterium *strains. It demonstrated a much higher level for MS941 than for WH320 (Figure 13). The *B. subtilis ywaD *gene encodes a double-zinc aminopeptidase whose function has not yet been characterized [80]. In general, aminopeptidases can participate in a wide range of biological processes, range from protein maturation or degradation to cell-cycle control. The higher secretion of YwaD in MS941 probably indicates, in general, a higher level of the cells in recycling small peptides that became available through the degradation of proteins/peptides in the culture medium by proteases and peptidases. These peptide fragments may at one hand serve as amino acid sources to sustain cell growth under nutrient limitations dominating the early fed-batch process. At the other hand they can play signaling roles in the initiation of different cellular processes such as competence development and sporulation.

### Stress responses

**Figure 14:**
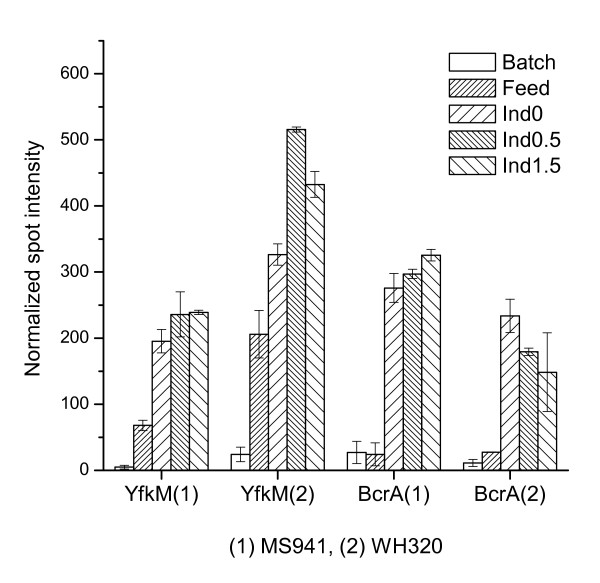
Expression profiles of two cell stress-related proteins. YfkM is a ThiJ/pfpI family protein; BcrA is the ATP-binding cassette of the BcrABC transporter for the transport of the antibiotic bacitracin.

In addition to the above described activation of the membrane-associated cleaning proteases HtrB which was implicated in cellular resistance to secretion stresses in the cell membrane/wall interphase, a number of stress-related proteins were found to be up-regulated in the high cell density fed-batch process, indicating increased cellular stresses with increased cell densities. Shown in Figure 14 are, as example, two proteins whose expression levels were significantly up-regulated under high cell density conditions.

A homologous protein of the σ^B^-dependent general stress protein YfkM of *B. subtilis *revealed a strongly elevated expression for both *B. megaterium *strains after entering the fed-batch phase and increased further as the cultivation proceeded. For WH320 an additional 58% increase of the YfkM level was observed after the induction of DsrS production. This coincided with the short-term high-level production and cytoplasmic accumulation of DsrS by WH320. YfkM is a ThiJ/pfpI family protein. Proteins from this family have a wide range of functions, but most importantly they possess chaperone and proteolytic activities that may contribute either to the correct folding of proteins or to the breakdown of misfolded or unnecessary proteins. YfkM is supposed to be a putative intracellular protease in *B. subtilis *and *B. cereus *[81,82]. Thus, the further increase of the YfkM level signaled perhaps an increased demand of the cells on such intercellular proteases to deal with stresses caused by aggregation of the large DsrS molecules in the cytoplasm. As a result, the yield of DsrS decreased after reaching a transient maximum volumetric activity (Table 1).

A homologue of the ATP-binding protein BcrA of *B. cereus *and *B. thuringiensis*, which is implicated in the transport of the antibiotic bacitracin, was identified in *B. megaterium *as strongly up-regulated in the late fed-batch phase under high cell density conditions for both strains (Figure 14). Bacitracin is a prominent inhibitor of cell wall biosynthesis and most active against Gram-positive bacteria [83]. BcrA is the ATP-binding cassette of the BcrABC transporter. This ABC transporter can mediate bacitracin-resistance through an active export of this antibiotic, an intrinsic resistance mechanism that generally possessed by the microbial antibiotic producers to become not susceptible to their own antibiotics [84,85]. Activation of the BcrABC transport system is found to be not restricted to bacitracin but also by general cell wall stresses induced by multiple cell wall-active antibiotics [86]. In our present study the elevated BcrA level at high cell densities might at least reflect high cell wall stresses related to the high cell density conditions for both *B. megaterium *strains.

## Conclusion

The two *B. megaterium *strains, MS941 and WH320, derived both from the same wild-type strain DSM319, demonstrated similar growth behavior considering biomass formation but were very different in the production of the heterologous dextransucrase under high cell density conditions. It seems that the fed-batch cultivation strategy employed in this study is more suitable for WH320 in respect of heterologous protein production.

Comparative proteomic analysis revealed changes in physiological responses of the bacterial cells to the given fed-batch cultivation conditions both within one strain and between the strains. The most significant discrepancies between the two strains were observed in the expression of enzymes involved in protein synthesis, protein secretion and in cell wall metabolism. For MS941 expression levels of many enzymes implicated in different stages of protein synthesis or in protein translocation across the cytoplasmic membrane were strongly reduced at high cell densities, accompanied with clear induction of a membrane-bound HtrA-type quality control protease Bmg4479. This was probably resulted from limitations in protein folding and secretion at the trans side of the cell membrane in the cells of MS941. Meanwhile, Protein Bmg4247 and Bmg0510, two peptidoglycan-binding proteins most likely involved in cell wall turnover, were also strongly induced and again only for MS941. As a result, cells of MS941 might have suffered from extensive cell wall turnover that ultimately led to severe cell lysis, as evidenced by the much higher extracellular protein concentration in the cultivation with MS941. The most significant differences between the two strains are summarized in Table 2. In order to understand what has caused such differences between the two *B. megaterium *strains, identification of mutated genes other than the known *lacZ *gene in the chemical mutant WH320 is necessary.

**Table 2:** Summary of some significant differences in protein expression levels.

Functional Category	Enzyme	Strain	
		
		MS941	WH320
YycFG TCS (Cell wall turnover, signal transduction)	Bmg4247	+++	-
	Bmg0510	+	-
	OppA	++	-
CssRS TCS (secretion stress)	Bmg4479	+	-
Protein secretion	SecA	RR	C
	FtsH	-	C
Sporulation	ORF1	+	-

Summary of some significant differences in protein expression levels between the two *B. megaterium *strains, MS941 and WH320, in the late fed-batch phase under high cell densities conditions.

"+++" very high expression; "++" high expression; "+" expression; "-" no expression detected; "RR" strongly reduced expression; "C" relatively constant expression

Taking together the impaired protein synthesis, increased secretion stresses and elevated cell lysis of MS941 it is tempting for us to speculate that already before the induction of DsrS production cells of MS941 were physiologically far away from maintaining a vegetative cell growth under the given high cell density conditions, let alone to produce a heterologous protein whose promoter system is actually optimal for gene expressions in the early exponential-growth phase [6]. In contrast, cells of WH320 seem to have remained more or less in a vegetative state under the same high cell density conditions that allowed a high-level volumetric production of DsrS but failed to secret it into the medium at all. It is worth to mention that until now most commercially available enzymes produced by *Bacillus *species are native proteins or homologous proteins of closely related *Bacillus *species. They are mainly hydrolytic enzymes, like amylases and proteases, which are naturally produced and secreted abundantly into the growth medium to degrade extracellular polymeric substrates for the purpose of sustaining cell growth under nutrient limitation conditions such as during stationary phase growth.

The carbon limited fed batch culture is the industrially well established technology to produce heterologous proteins in *E. coli*, yeasts and some *bacilli *as well. As exemplified in Figure 1 this approach is characterized by slow growth rates in the feeding phase to avoid oxygen starvation and excessive metabolic overflow of fermentative products and by induction of heterologous protein expression at high cell densities to achieve high volumetric productivities. The outcome of this study on congruent high cell density cultivations with two *B. megaterium *strains and the concomitant detailed proteome analysis clearly shows that fed batch cultivations are hardly applicable to the production of secreted heterologous proteins with *B. megaterium *for reasons given above. Therefore, other strategies that essentially provide an improved tuning of the expression and secretion machineries of the host cells are required.

Two important two-component systems, the CssRS TCS and the YycFG TCS, seem at least involved in the regulation of cell membrane- or cell wall-related cellular processes. Expression of the HtrA-type protein Bmg4479 is under the control of CssRS TCS in response to secretion stress in the cell membrane/wall interphase. YycFG TCS regulates, in addition to the peptidoglycan-binding protein Bmg4247 and Bmg0510 for cell wall turnover, also the OppA protein for the uptake of various extracellular oligopeptides that act as signal transducers for the cells to sense the environmental conditions. It is suggested that a whole apparatus of cell wall metabolic proteins is controlled by the YycFG system [54]. Thus, it would be very interesting to further characterize the functions of such two-component systems as well as genes controlled by them in *B. megaterium*. However, none of the histidine kinases and response regulators of any two-component systems were so far identified by 2-DE/MS based proteomic approaches, presumably due to too low amounts of these regulatory proteins. Therefore, in order to gain insight into the functions and regulatory mechanisms of such signal transduction systems, a comprehensive analysis at the transcriptional level is necessary. Study of global gene expressions both on the transcriptional level and the translational level will provide an overview of the bacterial physiology and responses to specific cultivation conditions.

## Materials and methods

### Bacterial strains and cultivation conditions

Two *B. megaterium *mutant strains, MS941 and WH320, were compared for the production of a heterologous dextransucrase (DsrS) in high cell density fed-batch cultivations. Both strains were derived from the wild-type strain DSM 319. MS941 is a defined Δ*nprM *mutant obtained by gene replacement of a major extracellular protease [65], whereas WH320 is a *lacZ *mutant constructed by chemical mutagenesis using ethyl methanesulfonate [41]. Both strains were transformed with the plasmid pMM1520 *dsrS *which carries the *dsrS *gene from *Leuconostoc mesenteroides *[6].

The glucose limited high cell density fed-batch cultivations as well as the analytical methods employed for the determinations of biomass, glucose and extracellular metabolites were described in detail previously [6] but with a minor modification, namely 1 ml of trace element solution and 0.3 g of MgSO_4_·H_2_O were added at 7-hour intervals during the feeding phase in addition to the feeding solution used. The cultivation conditions were controlled exactly the same for both strains, except the trace elements added during the batch phase, which was twice as much for MS941 as for WH320, since MS941 has been confirmed in the previous study to need more trace elements than WH320 to achieve comparable cell growth. DsrS expression was induced by adding xylose at a concentration of 0.5% w/v in the late fed-batch phase after the cultivations were carried out for 28.7 h and 28.2 h and the biomass concentrations reached 41.9 g/L and 44.7 g/L for MS941 and WH320, respectively.

### Preparation of intracellular and extracellular protein samples

Cell samples harvested at different time points during the high cell density cultivations were centrifuged at 6500 rpm (Sorvall RT 6000 B, DuPont) for 30 min at 4°C to obtain the supernatants and the cell pellets. The supernatants were precipitated overnight using 10% (w/v) trichloracetic acid (TCA), then centrifuged at 33,000 g and 4°C for 45 min. The pellets obtained were further washed 3 times with 10 ml of cold 96% ethanol (4°C), dried and stored at -80°C for two-dimensional gel electrophoretic (2-DE) analysis of the extracellular proteome (secretome). The cell pellets were washed twice with a buffer containing 0.1 M Tris HCl (pH 7.0), 10 mM DTT, 20 mM KCl, 5 mM MgCl_2 _and 1 mM EDTA, resuspended with a lysis buffer containing 7 M urea, 2 M thiourea, 4% (w/v) CHAPS, 1% (w/v) DTT, 0.8% (w/v) Pharmalyte™ pH 3–10, and 5 mM Pefabloc and disrupted by ultrasonication (Bandelin Sonopuls HD207 with power set at 60%) in an ice bath for 15 × 30 s with 60 s interval between each cycle. Cell debris was removed by centrifugation at 13,000 g and 4°C for 30 min. Raw protein extracts obtained were purified by phenol precipitation/acetone extraction according to the method described before [7]. Purified protein pellets were stored at -80°C for the 2-DE analysis of the intracellular (cell-associated) proteome.

### Proteomic analysis by two-dimensional gel electrophoresis

A rehydration buffer containing 7 M urea, 2 M thiourea, 0.5% (w/v) CHAPS, 1% (w/v) tritonX-100, 1% (w/v) ASB-14, 5 mM TCEP, 0.5% IPG buffer pH 4–7 and trace amount of bromphenol blue was used to resolve the purified intracellular or extracellular protein pellets. After centrifugation at 13,000 g and 4°C for 10 min the total protein concentration in the supernatant was determined using the PlusOne 2D-Quant kit (GE Healthcare) according to the manufacturer's instruction. 2-DE was carried out as described previously [7]. The first dimension isoelectric focusing (IEF) was carried out with 24 cm immobilized pH gradient (IPG) strips of pH 4–7 (GE Healthcare). Each IPG strip was loaded with 250 μg of proteins for intracellular proteomic analysis and 70 μg of proteins for extracellular proteomic analysis. The second dimension was run on self-cast 12.5% polyacrylamide gels. Duplicate or triplicate gels were used for each protein sample under reproducible experimental conditions.

Subsequently, 2-D PAGE gels were stained according to the improved fluorescent staining method described before [87] using ruthenium II tris-bathophenanthroline disulfonate (RuBPS), which was prepared according to the method described by Rabilloud *et al*. [88]. Gels were then scanned with the CCD based Fujifilm LAS-1000 image analyzer using the software Image Reader LAS-1000 Pro Version 2.5. The scanning parameters are set as follows: excitation wavelength 470 nm (Blue-SQW-LED), lens filter Y515-Di for fluorescence detection, exposure time 60 s, 14 bits images saved as .tif files. Computer image analysis of the gels were performed with the Phoretix 2D Advanced Software Version 2003.02 (Phoretix, Newcastle upon Tyne, UK) as described previously by Wang *et al*. [7]. Exactly same parameters were used for all gels for protein spot detection, background subtraction, matching and quantification. Minor manual adjustments during spot detection were performed when it was obviously necessary. User seeds were added to help spot matching between gels. Average gels were built from duplicate or triplicate gels of each sample. Average values of protein spot volume intensities and their corresponding standard deviations were calculated to characterize expression changes of proteins. To overcome electrophoretic variations protein spot intensity was defined as the normalized spot volume which is the ratio of the single spot volume to the total spots volumes on a 2-D gel. Normally only proteins showing more than 2-fold increase or decrease in expressions were considered to be up- or down-regulated.

### Protein identification by mass spectrometric analyses and the use of a specific *B. megaterium *protein database

Protein spots excised from 2-DE gels were subjected to tryptic digestion according to a method described previously [7] with some modifications. Briefly, protein spots were washed twice with Milli-Q water, dehydrated with acetonitrile, digested overnight with trypsin (sequencing grade modified, Promega Corp.) at 37°C, and desalted using the Montage ZipPlateC18 (Millipore Corp.) for parallel desalting of 96 digested protein samples. Subsequently, the tryptic peptides were analyzed by MALDI-TOF MS with a Bruker Ultraflex time-of-flight mass spectrometer (Bruker Daltonics GmbH, Germany) and, when necessary, by ESI-QqTOF MS/MS with a QTOF2 mass spectrometer (Micromass, Manchester, UK) as described before [89]. Peptide mass fingerprinting (PMF) was carried out using peptide masses obtained from MALDI-TOF MS analysis to search against a strain-specific *B. megaterium *protein database "bmegMEC.v2" with the MASCOT program licensed in-house. This protein database was developed from an improved genomic seuqence of *B. megaterium *wild-type strain DSM319, in comparison with the protein database "bmegMEC" reported before[13], which was derived from a low-coverage, unfinished genomic sequence. This improved genomic sequence was obtained by whole genome shotgun approach combing with fosmid walking and contains 288 contigs. Whenever necessary, partial amino acid sequences obtained from ESI-QqTOF MS/MS analysis were used for the verification of MALDI-TOF MS results.

## Competing interests

The author(s) declare that they have no competing interests.

## Authors' contributions

W.-D. Deckwer initiated and coordinated this study, and contributed to the preparation of this manuscript. W. Wang carried out the 2-DE/MS proteomic analysis and contributed mainly to the preparation of this manuscript. R. Hollmann carried out the high cell density cultivation experiments and related analysis, contributed to the preparation of this manuscript. All authors have read and approved the final manuscript.
